# Functional activity and connectivity signatures of ketamine and lamotrigine during negative emotional processing: a double-blind randomized controlled fMRI study

**DOI:** 10.1038/s41398-024-03120-6

**Published:** 2024-10-14

**Authors:** Marvin S. Meiering, David Weigner, Matti Gärtner, Luisa Carstens, Christian Keicher, Rita Hertrampf, Christian F. Beckmann, Maarten Mennes, Andreas Wunder, Anne Weigand, Simone Grimm

**Affiliations:** 1 Medical School Berlin, Berlin, Germany; 2 Department of Education and Psychology, Freie Universität Berlin, Berlin, Germany; 3 Charité Research Organisation GmbH, Berlin, Germany; 4 SBGneuro Ltd, Oxford, UK; 5 Translational Medicine and Clinical Pharmacology, Boehringer Ingelheim Pharma GmbH & Co. KG, Biberach an der Riss, Germany; 6 Department of Psychiatry and Psychotherapy, Charité, Universitätsmedizin Berlin, Corporate Member of Freie Universität Berlin and Humboldt-Universitiät Zu Berlin, Berlin, Germany; 7 Department of Psychiatry, Psychotherapy and Psychosomatics, University Hospital of Psychiatry, University of Zurich, Zurich, Switzerland

**Keywords:** Hippocampus, Molecular neuroscience

## Abstract

Ketamine is a highly effective antidepressant (AD) that targets the glutamatergic system and exerts profound effects on brain circuits during negative emotional processing. Interestingly, the effects of ketamine on brain measures are sensitive to modulation by pretreatment with lamotrigine, which inhibits glutamate release. Examining the antagonistic effects of ketamine and lamotrigine on glutamate transmission holds promise to identify effects of ketamine that are mediated through changes in the glutamatergic system. Investigating this modulation in relation to both the acute and sustained effects of ketamine on functional activity and connectivity during negative emotional processing should therefore provide novel insights. 75 healthy subjects were investigated in a double-blind, single-dose, randomized, placebo-controlled, parallel-group study with three treatment conditions (ketamine, lamotrigine pre-treatment, placebo). Participants completed an emotional face viewing task during ketamine infusion and 24 h later. Acute ketamine administration decreased hippocampal and Default Mode Network (DMN) activity and increased fronto-limbic coupling during negative emotional processing. Furthermore, while lamotrigine abolished the ketamine-induced increase in functional connectivity, it had no acute effect on activity. Sustained (24 h later) effects of ketamine were only found for functional activity, with a significant reduction in the posterior DMN. This effect was blocked by pretreatment with lamotrigine. Our results suggest that both the acute increases in fronto-limbic coupling and the delayed decrease in posterior DMN activity, but not the attenuated limbic and DMN recruitment after ketamine, are mediated by altered glutamatergic transmission.

## Introduction

The N-methyl-D-aspartate receptor antagonist ketamine has stimulated considerable research over the past two decades due to its rapid onset and robust, but transient, effects on symptomatology in patients with (treatment-resistant) major depressive disorder (MDD) and anxiety disorders [1–4]. On the molecular level, ketamine has been shown to act primarily on the glutamatergic system eliciting a cascade of neurobiological processes ultimately promoting neuroplasticity, primarily in the hippocampus and prefrontal cortex (PFC) [5–9].

Ketamine exerts a profound impact on emotion-related neural circuits. Some studies in healthy volunteers (HV) suggested that ketamine blunts responses in the amygdala and hippocampus during negative emotional processing [10, 11], whereas others reported enhanced reactivity of these regions [12, 13]. Notably, the latter two trials investigated negative emotional processing through tasks that combine emotional and cognitive components, which may contribute to these inconsistencies. Moreover, Lehmann et al. linked ketamine-induced deactivation of the anterior Default Mode Network (DMN) during negative emotional processing to the habitual employment of rumination and distraction, respectively [14]. Rumination describes the repetitive dwelling on potential causes and consequences of negative experiences and has been conceptualized as an important transdiagnostic risk factor for internalizing psychopathology [15–18]. Typically, an increased propensity for this dysfunctional coping mechanism is associated with hyperactivity and -connectivity of the DMN that primarily comprises the medial PFC (mPFC) and the posterior cingulate cortex (PCC) [19, 20]. Accordingly, over-recruitment of the DMN has been proposed to reflect the impaired ability to flexibly switch between task-positive and task-negative networks in response to environmental demands, resulting in perseverative negative thought processes [19, 21–26]. By showing that ketamine deactivates the DMN during negative emotional processing, Lehmann et al. provide evidence that ketamine may specifically target neuronal correlates of emotion regulation processes by restoring the balance and adaptive functioning of task-positive and task-negative brain networks [14].

Blunted limbic reactivity after ketamine has been linked to its antidepressant effects. While MDD is generally associated with amygdala and (para)hippocampal hyperactivity during negative emotional processing [27–32], ketamine has been shown to reduce amygdala and (para)hippocampal hyperactivity, which in turn is associated with symptom improvements [13, 33–35]. Also, the recruitment and interaction of distributed brain networks have been implicated in ketamine’s antidepressant mechanism of action. Although the DMN was not specifically mentioned in their work, Reed et al. reported reduced activity of core DMN nodes (mPFC and PCC) during negative emotional processing in MDD patients [13]. This finding corroborates previous findings of ketamine-induced attenuated DMN function in HV [14]. Furthermore, symptom improvement was found to be strongly correlated with increased fronto-limbic coupling after ketamine treatment in PTSD patients, suggesting enhanced top-down control via increased inhibitory effects of frontal on limbic regions [36].

Interestingly, imaging biomarkers of acute ketamine challenge are sensitive to modulation by pretreatment with compounds that modulate glutamate transmission. As previously demonstrated in HV, a single dose of lamotrigine attenuates ketamine-evoked BOLD and perfusion changes [37–42]. Given that lamotrigine inhibits glutamate release, it was concluded that the ketamine-induced brain changes were due to an increase in glutamate transmission.

However, until now, no study investigated the effects of lamotrigine pretreatment on the ketamine signal in the brain during negative emotional processing. In addition, longitudinal assessments of both the effects of ketamine and their modulation by lamotrigine may provide further insights, as the antidepressant effects of ketamine are most pronounced 24 h after administration, suggesting sustained adaptive changes in brain dynamics [1, 43]. Accordingly, the present study aimed to identify both acute and sustained effects of altered glutamate transmission by ketamine and lamotrigine on functional activity as well as connectivity (FC) during negative emotional processing in HV. More specifically, blunted responses in the amygdala and hippocampus by means of ketamine are hypothesized. Additionally, whole-brain approaches are employed to investigate the effects of ketamine on DMN recruitment and fronto-limbic coupling. Further, lamotrigine is hypothesized to attenuate the effects of ketamine on brain activity and connectivity. Given that rumination has been described as a transdiagnostic risk factor for internalizing psychopathology, a further exploratory aim of the study was to investigate whether ketamine modulates the association between DMN recruitment and rumination, as this may hold promise for linking findings in healthy participants to potential antidepressant mechanisms of ketamine.

## Materials and methods

### Participants

A total of 75 healthy male and female subjects aged 18–45 years underwent the fMRI procedures. The sample size was determined based on a power analysis for a one-way ANOVA assuming an alpha of 0.05, a beta of 0.8 and a large effect of f = 0.4 [44]. Exclusion criteria were history of or current psychiatric conditions, as determined by the SCID-5-CV at screening, a positive drug screen, previous participation in studies that used experimental paradigms employed here, prescribed psychotropic medication within 28 days prior to screening and non-prescription medication within 48 h prior to treatment visit. Further exclusion criteria were a history of relevant neurological diseases, migraine headaches, relevant medical conditions, MRI exclusion criteria, and pregnancy. All participants gave written consent to participate in the study, which was approved by the local ethics committee and registered at ClinicalTrials.gov (NCT04156035). No patients were included in this study.

### Study design

**Fig. 1:**
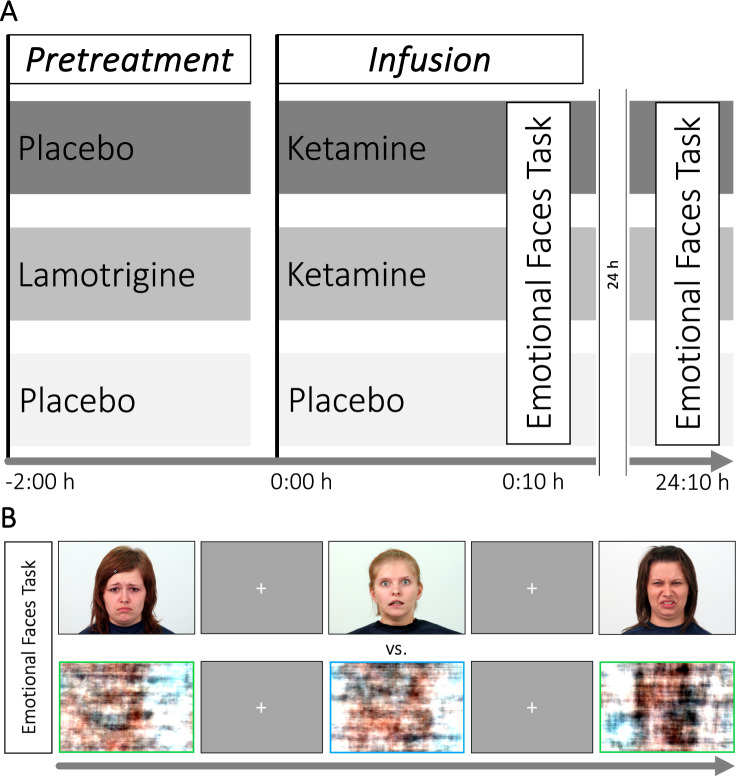
Design. **A** Study design and **B** experimental paradigm.

Details of the double-blind randomized controlled design of this study have been described in previous publications [41, 42]. Briefly, participants were randomly assigned to one of three treatment groups in a 1:1:1 ratio and were either pretreated with placebo and received a placebo infusion (placebo-placebo group, PP), pretreated with placebo and received a ketamine infusion (placebo-ketamine group, PK), or pretreated with lamotrigine and received a ketamine infusion (lamotrigine-ketamine group, LK, see Fig. 1A). All participants underwent two scanning sessions on two consecutive days. Before the first scanning session, pretreatment with an oral dose of 300 mg lamotrigine (LK) or matching placebo (PP, PK) occurred 2 h before the scanning procedures (−2:00 h with respect to infusion onset). During the first scanning session (acute), participants were intravenously administered racemic ketamine or placebo (ketamine dosage: 0.12 ± 0.003 mg/kg during the first minute followed by a continuous infusion of 0.31 mg/kg/h for approximately 55 min). Plasma concentration samples of lamotrigine were collected −3:00, −1:30, −1:00, −0:30, +0:55, and +2:00 h with respect to infusion onset. Plasma ketamine concentrations were collected immediately after scanning (+0:55 h from infusion onset). The emotional faces task commenced approximately 10 min after infusion onset. To investigate the delayed effects of ketamine on emotional processing, participants underwent the same scanning procedure without the drug treatment 24 h later. Additional experiments and modalities employed during this study have been reported elsewhere [41, 42].

### Emotional faces task

Using Presentation software (Neurobehavioral Systems, Inc, Berkeley, USA), participants were shown pictures of faces from the Warsaw Set of Emotional Facial Expression Pictures (WSEFEP; http://www.emotional-face.org). The task consisted of 12 blocks with 6 negative emotional faces (fear, disgust, sadness – randomized; affective condition, AC) and 12 blocks with scrambled pictures showing random color patterns with either a blue or a green frame (neutral condition, NC; see Fig. 1B). Within each block, 6 picture stimuli were shown for 3 s each. In sum, 72 negative faces of 24 actors (12 female, 12 male) were presented. After each stimulus, participants reported (via button press) the gender of the face (AC) or the color of the bounding box around the scrambled pictures (NC). Two matched sets of stimuli were used for the acute and post 24 h measurements to reduce the habituation of responses to repeated stimuli. The task lasted for 13 min. This version of the emotional faces task has been shown to specifically target limbic structures involved in negative emotional processing like the amygdala [45].

### Psychometric assessments

Psychotomimetic effects were assessed at both timepoints using the 5D Altered States of Consciousness Scale (5D-ASC; [46]) immediately after the scanning sessions. Mood was assessed using the Positive and Negative Affect Schedule (PANAS; [47, 48]) immediately before and after the scanning sessions. A subset of subjects participated in an optional follow-up study to obtain self-report measures of rumination, namely the German version of the Response Styles Questionnaire (RSQ; [49, 50]).

### Image acquisition and analyses

Imaging acquisition parameters are given in the supplement. Brain image preprocessing and analysis were carried out using FEAT (FMRI Expert Analysis Tool; [51–53]) version 6, as part of FSL (FMRIB’s Software Library; [54–56]). Information on the preprocessing and first-level analytical procedures for activity and psychophysiological interaction (PPI) metrics are given in the supplement.

#### Regions of Interest (ROI)

Regions were selected based on previous research on the effects of ketamine on emotional processing. The amygdalae and hippocampi were defined based on the Harvard-Oxford Subcortical Atlas. FSL featquery was used to extract BOLD percent signal changes (PSC) and contrast of parameter estimates (COPE). The 90^th^ percentile of PSC or COPE within all voxels of the respective ROI was used for subsequent statistical analyses.

#### Group-level

A General Linear Model using FSL FEAT was implemented to test for statistically significant activations and FC at the cluster level (voxel: z > 3.1, uncorrected; cluster: *p* < 0.05, FWE-corrected) using FSL Randomise, a permutation procedure, with 5000 iterations [57]. Local maximum Z statistics have been determined using parametrical t to z transformation. All whole-brain analyses were adjusted for the covariates age, sex as well as plasma concentration of ketamine and lamotrigine.

### ROI analyses

Statistical analyses were conducted in R, version 4.3.1 [58]. Extracted BOLD percent signal changes of the respective ROIs were tested for differences between groups (PK, LK, PP) using parametric ANCOVA for each time point independently, with HC3 correction to account for unequal variances as well as sex and age included as covariates. Additionally, ketamine and lamotrigine plasma concentrations at +0:55 h post infusion onset were included in the statistical models as covariates. Post hoc paired comparisons were conducted using permutation testing with 10,000 iterations. For reporting, an unadjusted significance level of α = 0.05 was assumed. To account for multiple comparisons, FDR-corrected *p*-values are reported. Standardized effect sizes of group differences are provided as Cohen’s d (including Hedge’s correction and assuming unequal variances). If a significant group difference was found, psychotomimetic effects (5D-ASC) and Negative Affect (PANAS) were included in the model as covariates to account for baseline negative mood and psychotomimetic side effects of ketamine. If neither variable explained a significant amount of variance in the data, they were dropped in favor of a more parsimonious model. Details regarding plasma concentration, 5D-ASC, and PANAS have been published elsewhere [41]. Finally, exploratory moderation analysis was conducted testing the group*rumination interaction term in a linear model. The code used to generate the results can be requested from the corresponding author.

## Results

A total of 73 subjects remained in the final sample for the acute and 72 for the delayed timepoint (for CONSORT flow diagram see Supplementary Fig. S1). Reasons for exclusion from fMRI analyses were structural abnormalities and exceeding of motion limit. The optional post hoc questionnaires were completed by 64 subjects from the initial sample, 62 remained for exploratory analyses after excluding participants with erroneous fMRI data quality. All results focus on the affective condition > neutral condition contrast of the paradigm (see supplement).

### Activity during negative emotional processing

#### ROI analyses

**Fig. 2:**
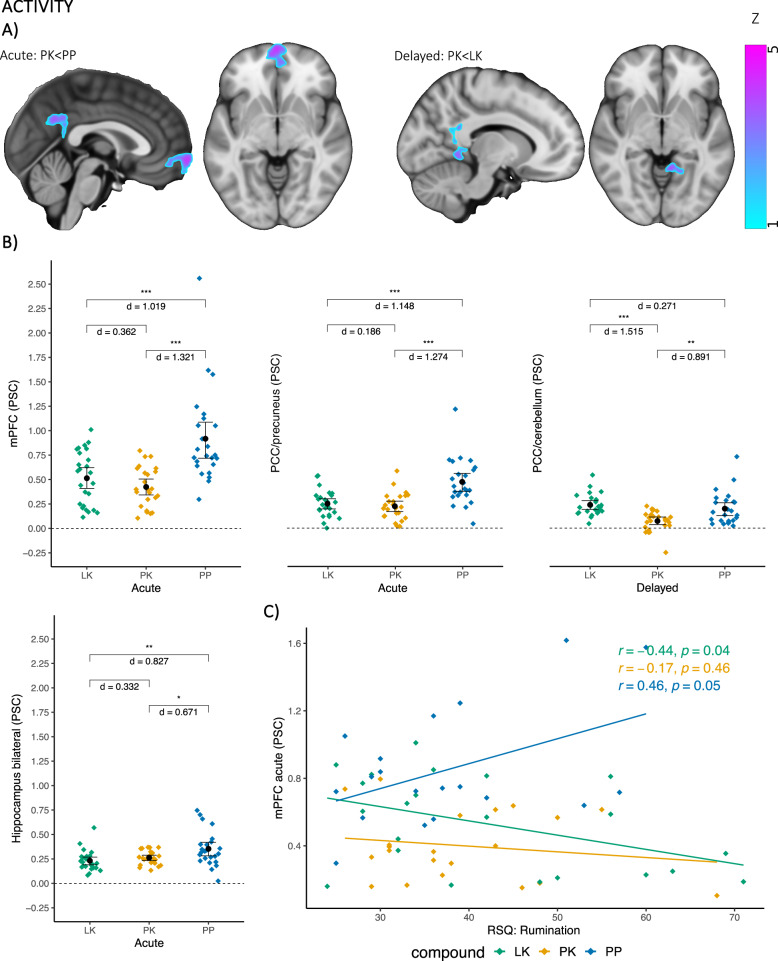
Activity results. **A** Whole-brain maps at the top, **B** Scatter plot of group differences in the middle, **C** Scatter plot of moderation analysis at the bottom, LK lamotrigine + ketamine, PK placebo + ketamine, PP placebo + placebo, mPFC medial prefrontal cortex, PCC posterior cingulate cortex; *p < 0.05; **p < 0.01; ***p < 0.001; PSC BOLD percent signal change, d Cohen’s *d*, RSQ response styles questionnaire, *r* Pearson correlation, *p* *p* value. Results are adjusted for age, sex, and plasma concentrations of ketamine and lamotrigine. Color code of the brain images reflects local z statistics. Error bars reflect 95% bootstrap confidence intervals (10,000 resamples).

At the acute time point, a significant difference between groups was found in the bilateral hippocampus (*F*(2, 66) = 3.312, *p* = 0.043, *p-FDR* = 0.141; see Table 1 and Fig. 2B, see Supplementary Tables S1 and S2 for all results including null findings and descriptive statistics), with post hoc paired comparisons showing significantly reduced reactivity in both the LK and PK groups compared to PP. Adding mood and psychotomimetic effects of ketamine as covariates did not explain additional variance in the data and did not change the results, so both were dropped from the final models. Moreover, no significant differences were observed for the hippocampus at the delayed time point (*F*(2, 65) = 0.751, *p* = 0.476, *p-FDR* = 0.630) and for the amygdala at both the acute (*F(*2, 66) = 2.170, *p* = 0.122, *p-FDR* = 0.250) as well as delayed time point (*F*(2,65) = 0.131, *p* = 0.878, *p-FDR* = 0.901).

**Table 1:** Post hoc paired comparisons.

ROI	LK-PK				LK-PP				PK-PP			
	*MD*	*d*	*p*	*p-FDR*	*MD*	*d*	*p*	*p-FDR*	*MD*	*d*	*p*	*p-FDR*
*Acute*												
Hippocampus bilateral	−0.030	−0.332	0.249	0.408	−0.122	−0.827	0.004	0.023	−0.092	−0.671	0.021	0.082
mPFC	0.090	0.362	0.209	0.365	−0.404	−1.020	< 0.001	< 0.001	−0.494	−1.320	<0.001	<0.001
PCC/precuneus	0.026	0.186	0.513	0.651	−0.223	−1.150	< 0.001	< 0.001	−0.249	−1.270	<0.001	<0.001
PPI: AMYr-aPFCr	−0.054	−1.010	< 0.001	< 0.001	0.018	0.481	0.096	0.225	0.072	1.300	<0.001	<0.001
*Delayed*												
Hippocampus bilateral	0.027	0.211	0.466	0.626	−0.018	−0.144	0.619	0.717	−0.045	−0.350	0.217	0.371
PCC/cerebellum	0.166	1.515	<0.001	<0.001	0.040	0.271	0.361	0.513	−0.127	−0.891	0.002	0.013

Results are adjusted for age, sex, and plasma concentrations of ketamine and lamotrigine.

*LK* lamotrigine + ketamine, *PK* placebo + ketamine, *PP* placebo + placebo, *MD* mean difference, *d* Cohens d, *p* permutation *p*-value, *p-FDR* false discovery rate corrected *p*-value, *mPFC* medial prefrontal cortex, *PCC* posterior cingulate cortex, *PPI* psychophysiological interaction, *AMYr* Amygdala right, *aPFCr* right anterior prefrontal cortex.

#### Whole-brain

At the acute time point, ketamine significantly reduced BOLD responses in the mPFC and PCC/precuneus compared to placebo (contrast: PK < PP, Fig. 2A and Table 2). At the delayed time point, pretreatment with lamotrigine increased BOLD responses in the PCC/cerebellum compared to ketamine (contrast: PK < LK; Table 2, Fig. 2A). No other significant differences were observed on the whole-brain level at the acute or the delayed time point. The PCC/precuneus and PCC/cerebellum clusters are spatially distinct and overlap only by 7 voxels (see Supplementary Fig. S2 for an overlay image). BOLD percent signal changes of the three clusters (mPFC, PCC/precuneus, PCC/cerebellum) were extracted and post hoc paired comparisons were performed on these measures. For the mPFC and PCC/precuneus clusters that emerged during infusion, significantly reduced reactivity was found in the PK and LK groups compared to PP, but no differences were observed between PK and LK (see Table 1 and Fig. 2B). For the PCC/cerebellum cluster that emerged 24 h later, reduced activity was found in the PK group compared to both the PP and LK groups (see Table 1 and Fig. 2B).

**Table 2:** Whole-brain results.

*Region*	*Contrast*	*Cluster size*	*p-FWE*	*Maximum Z*	*MNI coordinates (maximum Z voxel)*		
					x	y	z
*ACTIVITY*							
*Acute*							
mPFC	PK < PP	401	0.011	4.75	−6	64	−2
PCC/precuneus	PK < PP	261	0.029	4.75	−14	−42	30
*Delayed*							
PCC/cerebellum	PK < LK	278	0.033	4.13	−6	−48	−8
*CONNECTIVITY*							
*Seed: Amygdala right*							
aPFC right	PK > PP	417	0.007	4.15	34	54	28
Cerebellum left	PK > PP	204	0.027	4.15	−28	−54	−20
mCC left	PK > PP	176	0.034	4.96	−10	−18	34
SPL left	PK > PP	158	0.038	4.38	−24	−56	48
Central gyrus bilateral	PK > PP	151	0.040	4.06	−20	−28	48
*Seed: Hippocampus right*							
Central gyrus right	PK > PP	221	0.017	4.39	30	−30	60
pMTG right	PK > PP	174	0.026	4.96	66	−20	−6
pMTG left	PK > PP	163	0.030	4.33	−58	−28	0
Cerebellum bilateral	PK > PP	146	0.035	4.2	38	−76	−36
AG right	PK > PP	133	0.041	4.33	44	−48	52

*PK* placebo + ketamine, *PP* placebo + placebo, *LK* lamotrigine + ketamine, *mPFC* medial prefrontal cortex, *PCC* posterior cingulate cortex, *aPFC* anterior prefrontal cortex, *mCC* midcingulate cortex, *SPL* superior parietal lobe, *pMTG* posterior middle temporal gyrus, *AG* angular gyrus.

### PPI during negative emotional processing

**Fig. 3:**
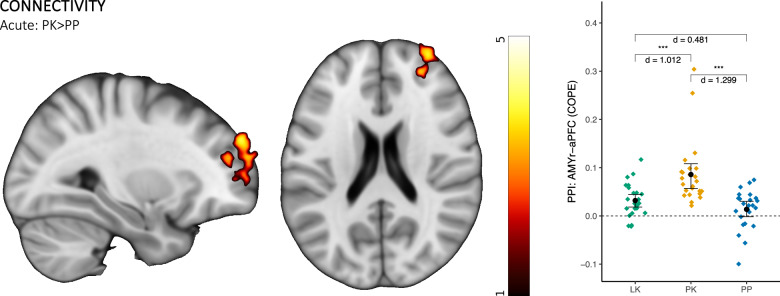
FC results. LK lamotrigine + ketamine, PK placebo + ketamine, PP placebo + placebo, PPI psychophysiological interaction; *p < 0.05; **p < 0.01; ***p < 0.001; d Cohen’s d, AMYr amygdala right, aPFC anterior prefrontal cortex, COPE contrast of parameter estimates. Results are adjusted for age, sex, and plasma concentrations of ketamine and lamotrigine. Color code of the brain images reflects local z statistics. Error bars reflect 95% bootstrap confidence intervals (10,000 resamples).

PPI analyses using the right amygdala as seed region revealed five clusters that showed significantly increased coupling during ketamine administration, with the right anterior prefrontal cortex (aPFC) as the only region located in the prefrontal area (Fig. 3 and Supplementary Fig. S3, Table 2). Post hoc paired comparisons of the increased coupling between the amygdala and the right aPFC revealed increased FC in the PK group compared to both the PP and LK groups (Table 1 and Fig. 3). In addition, using the right hippocampus as seed region, ketamine increased functional coupling during negative emotional processing at the acute time point in various regions, however, none of them were located in the prefrontal area (Supplementary Fig. S4 and Table 2). No significant FC differences between groups were observed for the amygdala and hippocampus at the delayed timepoint.

### Exploratory analyses

Exploratory moderation analyses revealed a significant group*rumination interaction for mPFC activity (*F*(2, 52) = 3.821, *p* = 0.028, *p-FDR* = 0.103). Simple slope analysis showed a positive association between rumination and mPFC recruitment for participants in the PP group, and a negative association for participants in the PK and LK groups (Fig. 2C). However, only the association in the LK group differed significantly from zero. Although the moderation analysis with the PCC/precuneus did not reach the threshold for statistical significance, a trend-level finding emerged (*F*(2, 52) = 2.845, *p* = 0.067, *p-FDR* = 0.189). Subsequent simple slope analysis revealed a pattern of associations comparable to the mPFC moderation analysis, with a positive correlation between rumination and PCC/precuneus reactivity in the PP group and no such association in the PK and LK groups (Supplementary Fig. S5). However, none of these simple slopes were statistically significant.

## Discussion

To our knowledge, this is the first study to examine not only the acute and delayed effects of a single dose of ketamine on both brain activity and FC during negative emotional processing, but also how these might be affected by inhibiting glutamate release using lamotrigine. Our results provide evidence that acute ketamine administration reduces hippocampal, mPFC, and PCC/precuneus reactivity during negative emotional processing, and increases coupling between the amygdala and the aPFC. The association between mPFC activity and rumination was significantly moderated by ketamine. Moreover, while lamotrigine attenuated the ketamine-induced increased fronto-limbic coupling at the acute timepoint, it had no such effects on hippocampal, mPFC, and PCC/precuneus activation. At the delayed time point, ketamine was found to significantly reduce responses in the PCC/cerebellum, which was blocked by pretreatment with lamotrigine.

The reduced hippocampal reactivity observed here further corroborates previous findings of decreased limbic reactivity after ketamine administration [11–13, 33–35, 59]. Although the hippocampus is primarily involved in memory and spatio-temporal processing, recent evidence suggests its involvement in prolonged negative emotional responses through rich projections from the amygdala, impairing the adaptive functioning of cells that, for example, encode the duration and boundaries of one event to another [60]. Accordingly, this impairment of hippocampal function might be a neural correlate of the inability to contain negative emotional responses into temporally distinct events, that are clearly distinguishable from others that do not share the same aversive qualities [60]. As a result, negative emotional responses cross the boundaries from one event to another, resulting in persistent experiences of negative affect, that are typically observed in internalizing disorders. The close relationship between the amygdala and the hippocampus is also evident by their relatively robust joint hyperactivity and connectivity during negative emotional processing in MDD [27–32, 61]. Furthermore, hippocampal reactivity during threat has been found to be positively associated with anxiety in HV [62]. As ketamine has also been shown to be an effective treatment for anxiety disorders, it may specifically target negative emotional processing at the circuit level, blunting hippocampal hyperreactivity in response to signals of potential threat [4]. Interestingly, pretreatment with lamotrigine resulted in blunted hippocampal reactivity similar to that seen in the ketamine group. Given that no modulatory effect of lamotrigine pretreatment was observed, we hypothesize that hippocampal blunting during ketamine infusion does not rely on changes in glutamatergic neurotransmission. However, several other factors could have contributed to this result. Unlike ketamine, which was administered intravenously at doses adapted to the body weight of each subject, lamotrigine was administered orally at a generic dose of 300 mg, regardless of body weight. Accordingly, lamotrigine metabolism may have been too heterogeneous between subjects to observe its inhibitory effects on the glutamatergic system. Despite the standard administration of a relatively high dose of lamotrigine (300 mg), the optimal dose to robustly observe the hypothesized effects on the glutamatergic system in combination with ketamine is still unknown and may fall outside the range used here. To account for this limitation, plasma concentrations of lamotrigine (and ketamine) were included in the models to control for the effects of compound bioavailability on the brain signal. This is a particular strength of this study, as none of the previous studies investigating the interaction of ketamine and lamotrigine on functional brain changes have accounted for this source of heterogeneity.

Although amygdala reactivity also showed a nominal decrease of moderate magnitude, the difference did not reach statistical significance. One reason for this null finding may be the characteristics of the sample. Our sample consisted of HV, who generally show lower amygdala reactivity to negative emotional stimuli compared to MDD patients [32]. Therefore, ceiling effects may have prevented ketamine from exerting its blunting effects on amygdala function in our sample. Neuroimaging results regarding effects of ketamine on amygdala reactivity during negative emotional processing are scarce. Only two studies were able to provide support for the hypothesis of blunted amygdala responses in HV [10, 11], whereas several studies were able to support this notion in patients [13, 33–35, 59].

At a whole-brain level, reduced activation was seen in the PCC/precuneus and mPFC during infusion and in the PCC/cerebellum 24 h after infusion. Both nodes (mPFC, PCC) form the core network of the DMN and have been implicated in the pathophysiology of MDD, especially in the context of rumination and treatment resistant depression [21, 23, 63–66]. Specifically, increased activity and FC within the DMN have been linked to rumination. [19, 20]. Accordingly, it has been proposed that over-recruitment of the DMN reflects an impaired ability to flexibly switch between task-positive and task-negative networks in response to environmental demands, resulting in perseverative negative thought processes [19, 21–26]. Our findings suggest that ketamine may act on the over-recruitment of the DMN during negative emotional processing. This is also supported by the results of the exploratory moderation analysis, which showed that individuals who reported greater rumination also showed greater reactivity of the anterior DMN (mPFC) in the placebo group but not in the two groups that received ketamine. We therefore hypothesize that individuals prone to rumination may benefit more from ketamine by restoring normative DMN function, although this needs to be supported by further research in MDD patients. Our finding aligns with a previous report of decreased anterior DMN reactivity during negative emotional processing 24 h after ketamine treatment in HV [14]. In that study it was demonstrated that subjects employing distraction, an adaptive coping mechanism conceptualized diametrically to rumination, to a lesser extent showed improved downregulation of DMN activity after ketamine, thereby suggesting that these individuals benefit more strongly from ketamine. Similar to the finding of diminished hippocampal reactivity, pretreatment with lamotrigine did not block the acute ketamine-induced attenuation of the DMN. However, pretreatment with lamotrigine affected neural responses in a PCC/cerebellum cluster 24 h post infusion, with significantly lower reactivity in the ketamine group compared to both the lamotrigine and the placebo group. It has been proposed that the PCC comprises three distinct functional subregions, the dorsal PCC (dPCC), the ventral PCC (vPCC) and the retrosplenial cortex (RSC; [67]). The vPCC and dPCC correspond to the PCC/precuneus cluster, which showed reduced reactivity during infusion. In contrast, the PCC/cerebellum cluster, where sustained effects of ketamine were shown, closely follows the outline of the RSC. Despite their dissociable location, the PCC and RSC are congruently connected to the DMN [68], suggesting that ketamine may exert time-dependent differential effects on DMN nodes. Considering that peak plasma concentrations occured between 1.3 and 4.5 h following administration and that lamotrigine’s half-life is between 15 and 30 h [69, 70], it can be speculated that the acute effects of ketamine are not mediated by the glutamatergic system and involve both the anterior and posterior DMN nodes, whereas the delayed effects of ketamine are mediated by the glutamatergic system and are restricted to the posterior DMN, particularly the RSC.

As already demonstrated by previous studies in both patients and HV, ketamine increased fronto-limbic coupling during negative emotional processing [12, 36]. A wealth of evidence has linked the interaction of frontal and limbic regions to emotion regulation, suggesting a central role for the PFC in the inhibitory top-down regulation of (negative) affect [71–75]. In this context, decreased fronto-limbic coupling during negative emotional processing has been shown to reflect the impaired ability of the PFC to exert inhibitory control over amygdala responses associated with MDD [76–78]. The ketamine-induced increased fronto-limbic coupling found in our study may point to improvements in these regulatory processes. Emotion regulation strategies can be placed in a framework spanning from explicit and controlled to implicit and automatic strategies [79]. In this framework, strategies which include the conscious goal to modulate emotions would be termed explicit, whereas implicit approaches do not involve this conscious goal. The controlled versus automatic dimension is defined orthogonal to the former, placing strategies on a continuum from purposefully and consciously used mechanisms to modulate emotional responses (controlled) to automatic mechanisms of emotion regulation. In the emotional faces viewing paradigm used in this study, subjects were instructed to judge the gender of the persons depicted without any instructions to modulate affect during the task. Accordingly, our task falls into the implicit and automatic domain, suggesting that ketamine may improve these regulatory processes. Interestingly, increased fronto-limbic coupling was significantly attenuated by pretreatment with lamotrigine. Therefore, it seems conceivable that ketamine-induced improved fronto-limbic coupling could be mediated by increased glutamatergic neurotransmission. Although the coupling between the amygdala and the PFC increased significantly, no regulatory effect on amygdala reactivity was found, making these conclusions preliminary. Furthermore, lamotrigine not only inhibits glutamate release, but has various effects such as the blockade of voltage-gated sodium channels and reduction of calcium currents, resulting in a reduction of neuronal excitability [80]. It also modulates GABA activity and has antioxidant effects [81, 82]. Therefore, its interaction with ketamine may be more complex and needs to be investigated in future studies.

### Limitations

First, the statistical approach to effective connectivity (PPI) used here is correlational in nature and therefore does not allow conclusions to be drawn about the direction of the fronto-limbic relationship. Increased top-down inhibitory control exerted by frontal regions on limbic structures has been hypothesized here, however, in the ketamine literature on negative emotional processing only a single study has employed adequate statistical methods to shed light on this question [36].

Although our results are consistent with the effects of ketamine observed in patient samples, the present results cannot be generalized to these populations. As shown by Reed et al., employing the same paradigm in patients and HV can yield very different results [13, 34]. The field is just beginning to unravel the complex underpinnings of neural correlates of psychopathology and their modulation by ketamine, and much work is still needed to reach definite conclusions.

Although imaging studies of ketamine are becoming increasingly available, the number of adequately powered studies is still quite limited, particularly in the area of negative emotional processing. The challenge of sufficient statistical power is also evident in our results, with only the whole-brain and FC statistics surviving a rigorous FDR correction. However, the present study, with a sample size of 25 subjects per group, is a positive step forward, given the relatively small sample sizes in previous studies, often involving 15 subjects or less [10, 35, 36, 59].

## Conclusion

This is the first study investigating not only the acute and delayed effects of ketamine on both brain activity and FC during negative emotional processing, but also its modulation by pretreatment with lamotrigine. Our results suggest that ketamine acutely acts on several brain systems involved in negative emotional processing: it decreases the reactivity of limbic circuits, ameliorates over-recruitment of the DMN, and improves fronto-limbic coupling.

Furthermore, our results suggest that both the acute effects of ketamine on fronto-limbic coupling and the sustained effects on PCC/cerebellum reactivity 24 h later are mediated by altered glutamatergic transmission. Further research in patients and HV is needed to confirm our findings and to improve our understanding of the underlying principles of how ketamine works in the brain.

## Supplementary information

Supplemental material

## Data Availability

All data generated or analyzed during this study are included in this article. Further enquiries can be directed to the corresponding author.
