# Correction: Functional activity and connectivity signatures of ketamine and lamotrigine during negative emotional processing: a double-blind randomized controlled fMRI study

**DOI:** 10.1038/s41398-024-03191-5

**Published:** 2024-12-04

**Authors:** Marvin S. Meiering, David Weigner, Matti Gärtner, Luisa Carstens, Christian Keicher, Rita Hertrampf, Christian F. Beckmann, Maarten Mennes, Andreas Wunder, Anne Weigand, Simone Grimm

**Affiliations:** 1 Medical School Berlin, Berlin, Germany; 2 Department of Education and Psychology, Freie Universität Berlin, Berlin, Germany; 3 Charité Research Organisation GmbH, Berlin, Germany; 4 SBGneuro Ltd, Oxford, UK; 5 Translational Medicine and Clinical Pharmacology, Boehringer Ingelheim Pharma GmbH & Co. KG, Biberach an der Riss, Germany; 6 Department of Psychiatry and Psychotherapy, Charité, Universitätsmedizin Berlin, Corporate Member of Freie Universität Berlin and Humboldt-Universitiät Zu Berlin, Berlin, Germany; 7 Department of Psychiatry, Psychotherapy and Psychosomatics, University Hospital of Psychiatry, University of Zurich, Zurich, Switzerland

**Keywords:** Hippocampus, Molecular neuroscience

Correction to: *Translational Psychiatry* 10.1038/s41398-024-03120-6, published online 14 October 2024

Table 1 and Table 2 in the Article were not numbered correctly. Table 1 should depict the ROI results whereas Table 2 should depict the whole-brain results.

The original article has been corrected.

