# Supplementary material for: Functional activity and connectivity signatures of ketamine and lamotrigine during negative emotional processing: a double-blind randomized controlled fMRI study
Source: Transl Psychiatry. 2024 Oct 14;14:436. doi: 10.1038/s41398-024-03120-6 (PMC11479267; doi:10.1038/s41398-024-03120-6)
Supplement: Supplementary file 1 — Supplemental material [file 41398_2024_3120_MOESM1_ESM.pdf]

Supplemental Material:

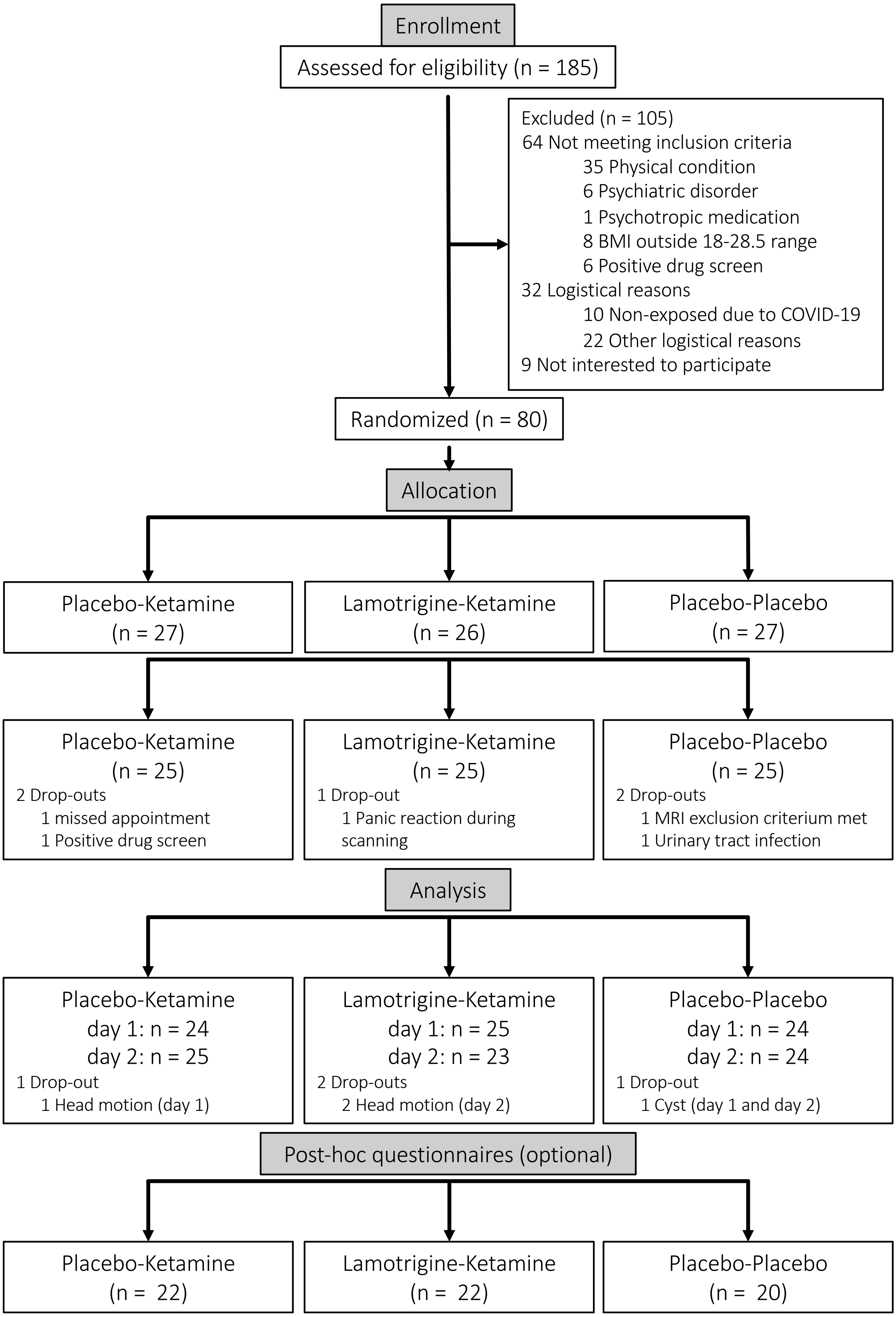

Figure S1. CONSORT flow diagram.

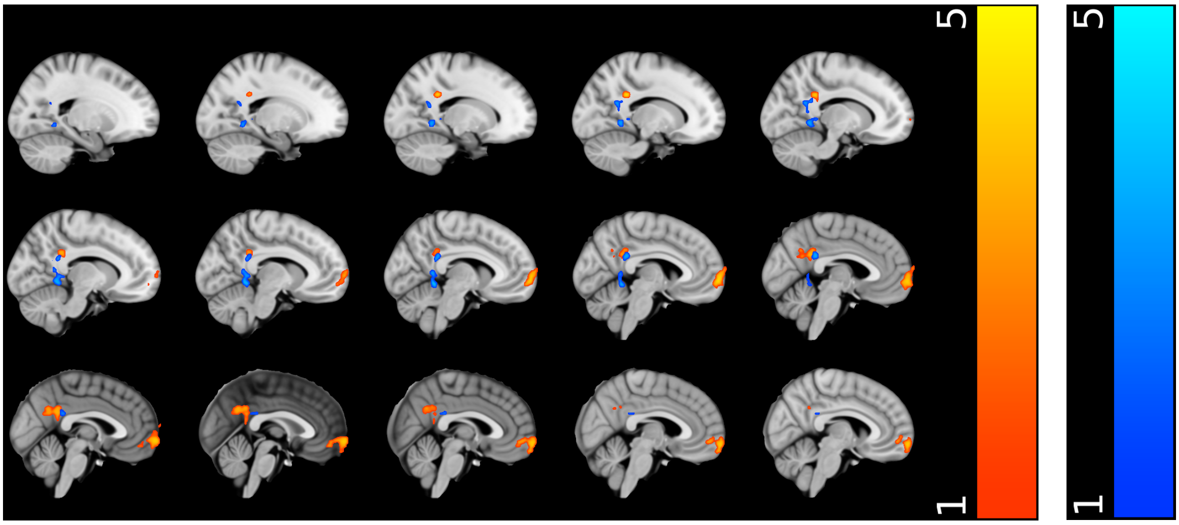

Figure S2. Significantly deactivated cluster at the acute (red; PK<PP) and delayed (blue; PK<LK) timepoint. Color bar depicts Z values.

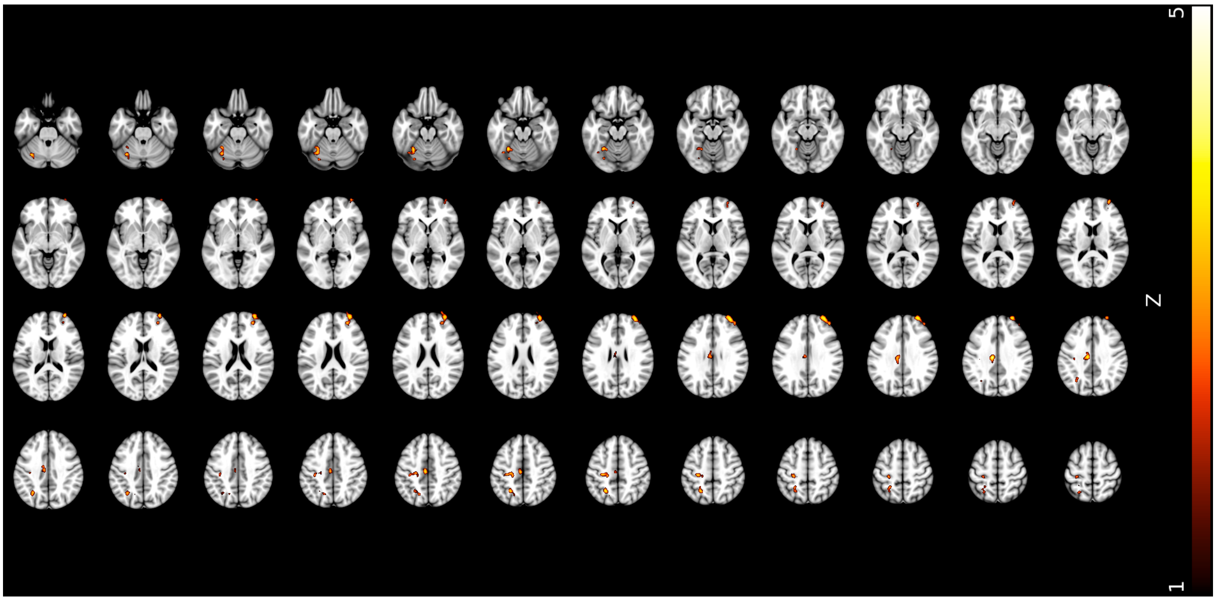

Figure S3. Psychophysiological interaction analysis: whole brain results (Seed: right amygdala). Color bar depicts Z values.

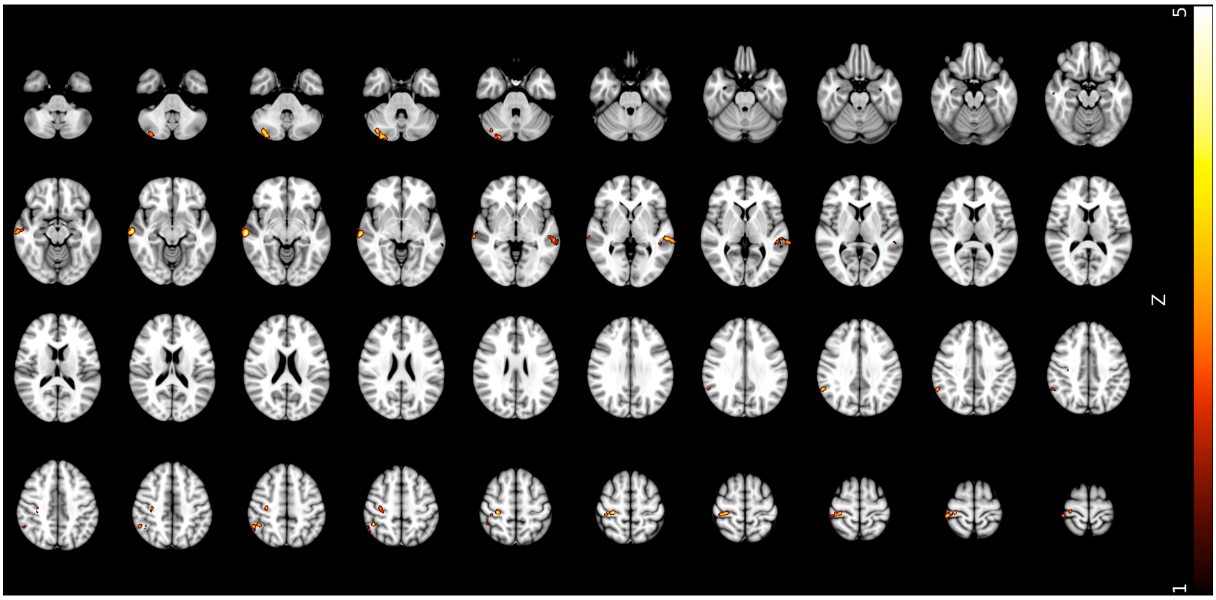

Figure S4. Psychophysiological interaction analysis: whole-brain results (Seed: right hippocampus).

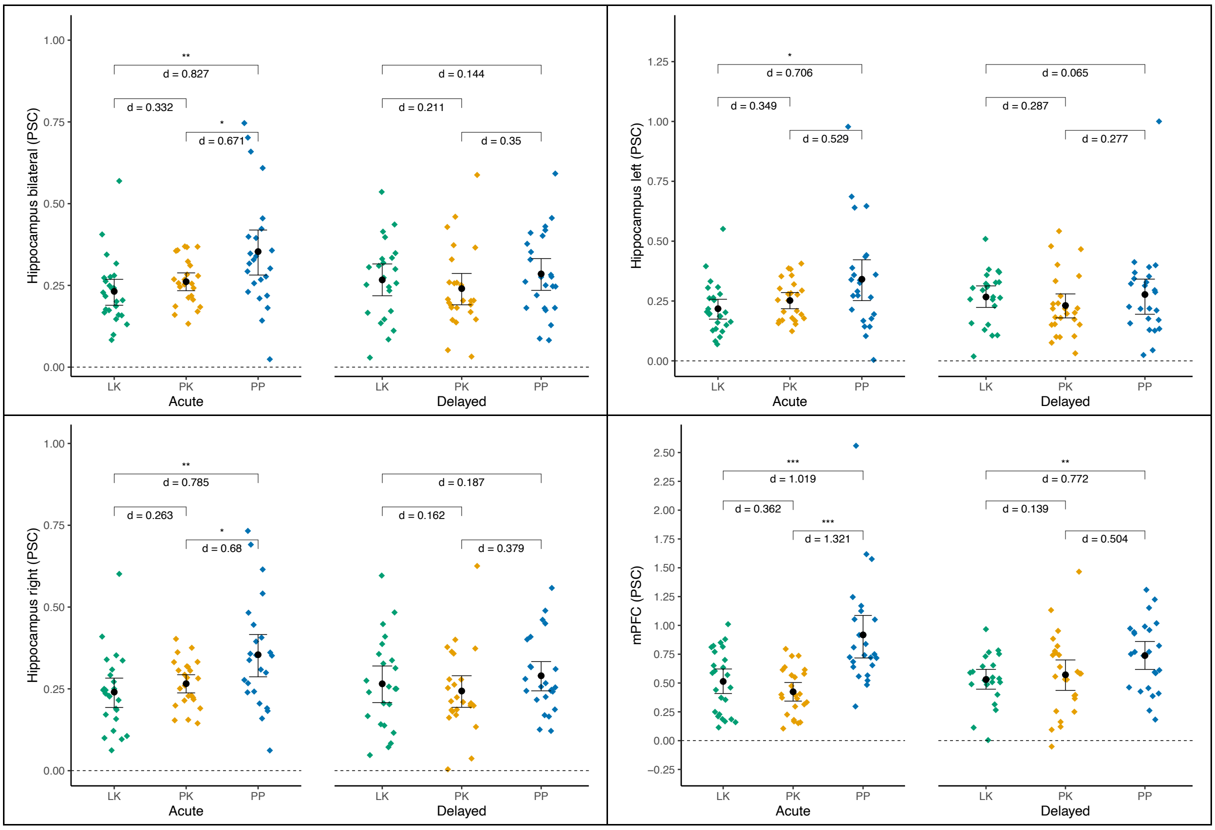

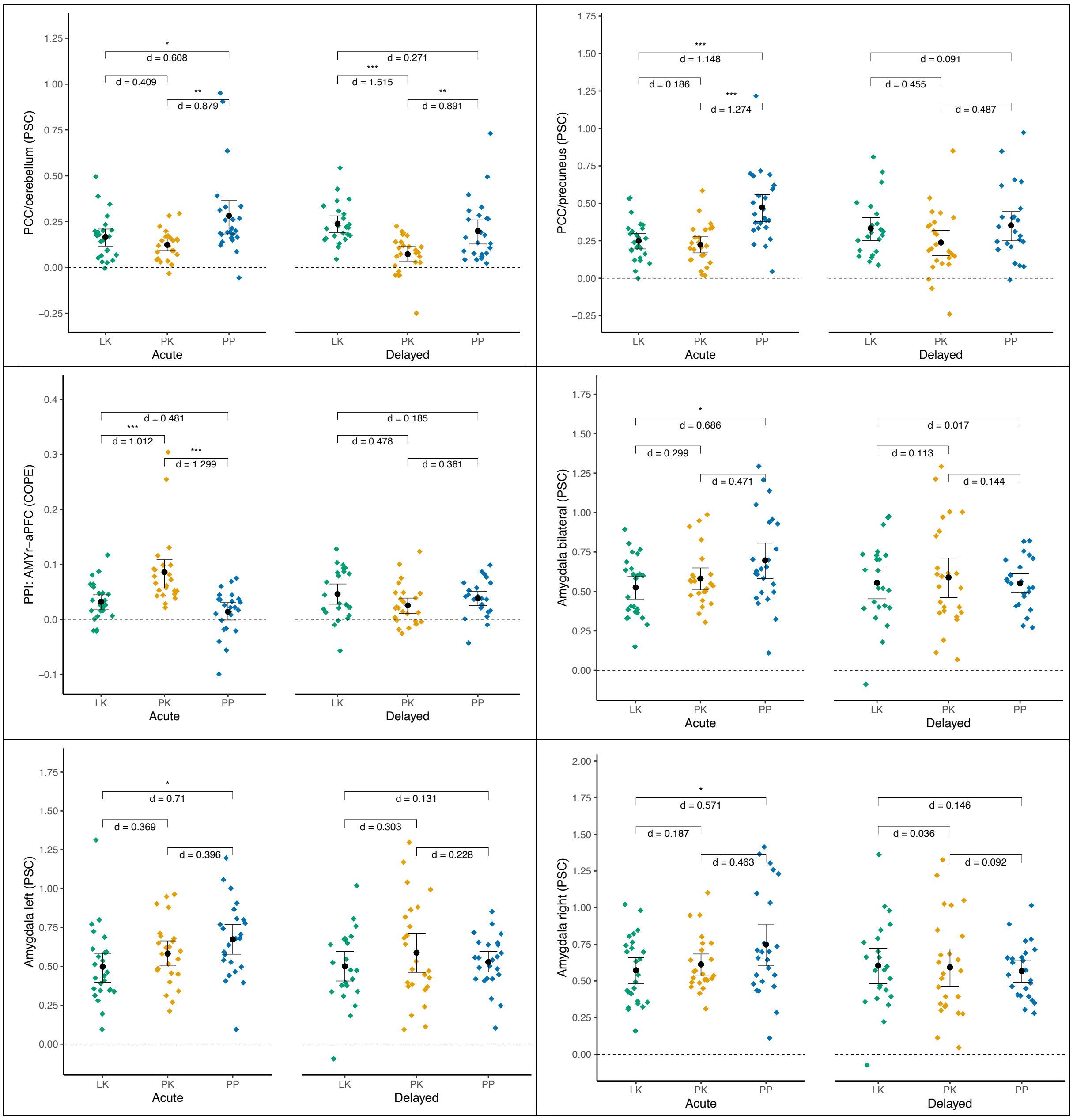

*Figure S4. Activity: Scatterplots of selected ROIs. ROIs of the mPFC, PCC and aPFC were derived from the significant clusters which emerged in the whole-brain analysis. d, Cohen's d; ns, not significant; *, p < 0.05; **, p < 0.01; ***, p > 0.001; PSC, BOLD percent signal change; LK, lamotrigine + ketamine; PK, placebo + ketamine; PP, placebo + placebo; AMYr-aPFC, functional connectivity between right amygdala and right anterior prefrontal cortex; COPE, contrast of parameter estimates. Error bars represent 95% confidence intervals (bootstrap: 10000 resamples).*

Table S1. Descriptive statistics and ANCOVA

ROI		*M (SD)*		*F*	*p*	*p-FDR*
Acute	LK (25)	PK (24)	PP (24)	df (2, 66)		
Amygdala bilateral	0.525 (0.191)	0.581 (0.178)	0.696 (0.290)	2.170	0.122	0.250
Amygdala right	0.573 (0.232)	0.613 (0.190)	0.749 (0.361)	1.618	0.206	0.365
Amygdala left	0.498 (0.244)	0.583 (0.207)	0.673 (0.242)	2.345	0.104	0.230
Hippocampus bilateral	0.232 (0.103)	0.261 (0.071)	0.353 (0.177)	3.312	0.043	0.141
Hippocampus right	0.240 (0.116)	0.266 (0.072)	0.354 (0.166)	2.856	0.065	0.189
Hippocampus left	0.218 (0.107)	0.252 (0.085)	0.341 (0.218)	2.644	0.079	0.202
mPFC	0.514 (0.277)	0.424 (0.205)	0.918 (0.478)	9.462	< 0.001	< 0.001
PCC/precuneus	0.250 (0.135)	0.224 (0.138)	0.473 (0.235)	7.955	< 0.001	< 0.001
PCC/cerebellum	0.166 (0.119)	0.124 (0.081)	0.282 (0.236)	3.574	0.034	0.116
PPI: AMYr-aPFCr	0.032 (0.034)	0.086 (0.066)	0.014 (0.040)	6.639	0.002	0.013
Delayed	LK (23)	PK (25)	PP (24)	df (2, 65)		
Amygdala bilateral	0.555 (0.258)	0.589 (0.327)	0.551 (0.157)	0.131	0.878	0.901
Amygdala right	0.605 (0.302)	0.593 (0.339)	0.567 (0.189)	0.130	0.879	0.901
Amygdala left	0.500 (0.239)	0.588 (0.325)	0.528 (0.171)	0.419	0.659	0.730
Hippocampus bilateral	0.267 (0.122)	0.240 (0.126)	0.285 (0.126)	0.751	0.476	0.630
Hippocampus right	0.265 (0.141)	0.243 (0.126)	0.290 (0.116)	1.104	0.368	0.513
Hippocampus left	0.267 (0.113)	0.231 (0.131)	0.277 (0.190)	0.517	0.599	0.717
mPFC	0.531 (0.216)	0.571 (0.345)	0.739 (0.306)	2.763	0.071	0.194
PCC/precuneus	0.333 (0.191)	0.238 (0.217)	0.353 (0.247)	1.373	0.261	0.420
PCC/cerebellum	0.238 (0.113)	0.072 (0.102)	0.199 (0.169)	9.688	< 0.001	< 0.001
PPI: AMYr-aPFCr	0.046 (0.046)	0.025 (0.037)	0.038 (0.033)	1.089	0.343	0.511

Notes: LK, lamotrigine + ketamine; PK, placebo + ketamine; PP, placebo + placebo; df, degrees of freedom; p-FDR, false discovery rate corrected p-value; mPFC, medial prefrontal cortex; PCC, posterior cingulate cortex; PPI, psychophysiological interaction; AMYr, Amygdala right; aPFCr, right anterior prefrontal cortex. Results are adjusted for age, sex, and plasma concentrations of ketamine and lamotrigine. Number in parentheses represent sample size and SD, respectively.

.

Table S2. Post-hoc paired comparisons

ROI	LK-PK				LK-PP				PK-PP			
	MD	*d*	*p*	*p-FDR*	*MD*	*d*	*p*	*p-FDR*	*MD*	*d*	*p*	*p-FDR*
Acute												
Amygdala bilateral	-0.056	-0.299	0.299	0.472	-0.171	-0.686	0.016	0.069	- 0.115	-0.471	0.102	0.230
Amygdala right	-0.040	-0.187	0.512	0.651	-0.176	-0.571	0.045	0.142	-0.136	-0.463	0.109	0.235
Amygdala left	-0.085	-0.369	0.202	0.365	-0.176	-0.710	0.014	0.064	-0.091	-0.396	0.170	0.332
Hippocampus bilateral	-0.030	-0.332	0.249	0.408	-0.122	-0.827	0.004	0.023	-0.092	-0.671	0.021	0.082
Hippocampus right	-0.026	-0.263	0.369	0.513	-0.114	-0.785	0.006	0.033	-0.089	-0.680	0.020	0.082
Hippocampus left	-0.034	-0.349	0.222	0.372	-0.123	-0.706	0.012	0.062	-0.089	-0.529	0.066	0.189
mPFC	0.090	0.362	0.209	0.365	-0.404	-1.020	< 0.001	< 0.001	-0.494	-1.320	< 0.001	< 0.001
PCC/precuneus	0.026	0.186	0.513	0.651	-0.223	-1.150	< 0.001	< 0.001	-0.249	-1.270	< 0.001	< 0.001
PCC/cerebellum	0.042	0.409	0.156	0.312	-0.115	-0.608	0.029	0.103	-0.158	-0.879	0.001	0.007
PPI: AMYr-aPFCr	-0.054	-1.010	< 0.001	< 0.001	0.018	0.481	0.096	0.225	0.072	1.300	< 0.001	< 0.001
Delayed												
Amygdala bilateral	-0.034	-0.113	0.699	0.764	0.004	0.017	0.949	0.949	0.037	0.144	0.621	0.717
Amygdala right	0.012	0.036	0.901	0.912	0.037	0.146	0.608	0.717	0.026	0.092	0.750	0.805
Amygdala left	-0.088	-0.303	0.305	0.472	-0.028	-0.131	0.647	0.727	0.060	0.228	0.435	0.595
Hippocampus bilateral	0.027	0.211	0.466	0.626	-0.018	-0.144	0.619	0.717	-0.045	-0.350	0.217	0.371
Hippocampus right	0.022	0.162	0.572	0.700	-0.025	-0.187	0.516	0.651	-0.047	-0.379	0.181	0.345
Hippocampus left	0.036	0.287	0.322	0.489	-0.010	-0.065	0.848	0.891	-0.046	-0.277	0.351	0.513
mPFC	-0.041	-0.139	0.638	0.727	-0.208	-0.772	0.013	0.063	-0.167	-0.504	0.076	0.201
PCC/precuneus	0.095	0.455	0.120	0.250	-0.021	-0.091	0.756	0.805	-0.115	-0.487	0.089	0.221
PCC/cerebellum	0.166	1.515	< 0.001	< 0.001	0.040	0.271	0.361	0.513	-0.127	-0.891	0.002	0.013
PPI: AMYr-aPFCr	0.020	0.478	0.094	0.225	0.008	0.185	0.526	0.654	-0.013	-0.361	0.203	0.365

Notes: LK, lamotrigine + ketamine; PK, placebo + ketamine; PP, placebo + placebo; MD, mean difference; d, Cohens d; p, permutation p-value; p-FDR, false discovery rate corrected p-value; mPFC, medial prefrontal cortex; PCC, posterior cingulate cortex; PPI, psychophysiological interaction; AMYr, Amygdala right; aPFCr, right anterior prefrontal cortex. Results are adjusted for age, sex, and plasma concentrations of ketamine and lamotrigine.

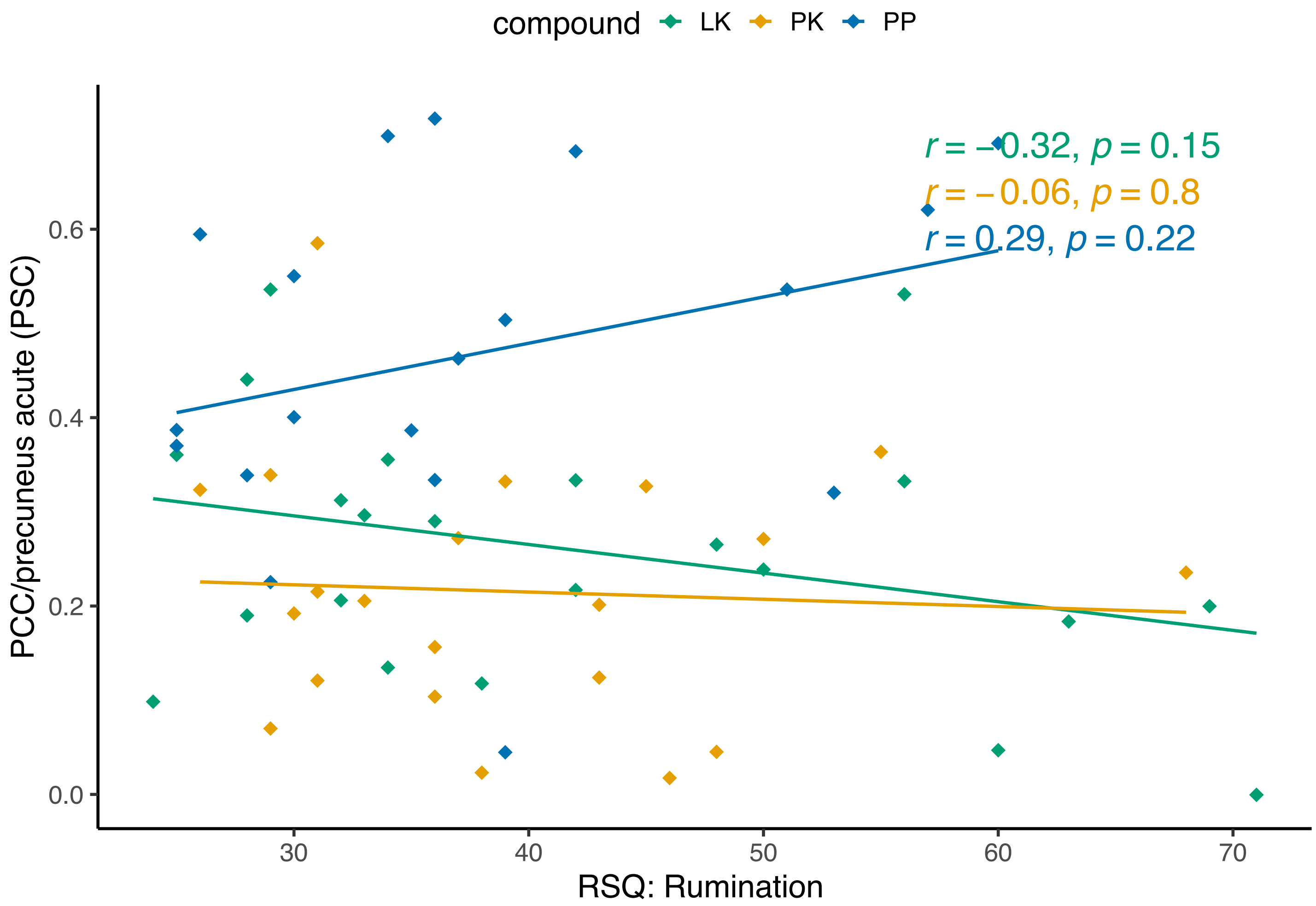

Figure S5. Moderation analysis: PCC/precuneus activity.

Sample Size

Power analysis for a one-way ANOVA with 3 groups was conducted in G*Power (Faul et al. 2013) to determine a sufficient sample size using an alpha of 0.05, a power of 0.80, and a large effect size of f = 0.4. This effect size (averaged for acute and delayed effects of ketamine as both are primary endpoints) was based on previously reported effect sizes (d = 0.815 for acute effects and d = 0.776 for delayed ketamine effects, Abdallah et al. 2018), showing a significant increase in prefrontal global connectivity during infusion and at 24-h posttreatment as compared to placebo. The sample size needed with this effect size was N = 66 (22 subjects per group). Accounting for a drop-out rate of ~15 %, the estimated total sample size was N = 75 (25 subjects per group). Screening for the trial was stopped once a sufficient number of subjects had been measured and controlled for dropouts due to excessive head movement in the scanner and/or headaches. Total study duration was from March 05, 2020 to December 10, 2020.

Randomization

Randomization, enrollment of participants, and assignment to interventions was conducted by the Charité Research Organisation (CRO), GmbH. After written informed consent, subjects meeting all in-/exclusion criteria were randomized at baseline to one of three experimental conditions (lamotrigine + ketamine, placebo + ketamine, placebo). The three treatments were allocated 1:1:1 randomization. Each subject received only one single dose of blinded combined study medication (lamotrigine + ketamine, placebo + ketamine or placebo) in the sequence according to randomization administered by the site staff. Randomization numbers were assigned in ascending, sequential order to eligible subjects. Additional numbers were used in case of replacements being needed. The investigator documented the randomization number in the eCRF. The randomization list was kept in safe and confidential custody. Only personnel not involved in the study had access to the list.

Blinding

For the conduct of this study, the study drugs as well as matching placebos (lamotrigine + ketamine, placebo + ketamine or placebo) were administered in a double-blind fashion at the site. Doses were prepared by an unblinded member of the study team. This unblinded member was not involved in any study assessments. The identity of the treatments was concealed by the use of study drugs that were all identical in packaging, labeling, schedule of administration, appearance,

and odor. Only the unblinded site team had access to the unblinded randomization list. Subjects remained blinded to study treatment throughout the study. Anyone who was involved in subject-related assessments was blinded with regards to the treatment assigned.

Harms

Safety assessments included vital signs, physical examinations, ECGs, standard clinical laboratory evaluations (hematology, blood chemistry, and coagulation), adverse events and serious adverse event monitoring.

Adverse events

No severe adverse events occurred. At screening appointments, a total of n = 3 minor pretreatment adverse events were noted: Respectively one participant was suffering from a skin rash, herpes labialis and a hordeolum.

Vital signs

Vital signs included pulse rate, systolic and diastolic blood pressure and body temperature. The body temperature was measured in the ear. Vital signs could be recorded at any time, if medically imperative for clarification of clinical signs and symptoms. For n = 35 participants abnormal vital signs were reported; body temperature, blood pressure or pulse rate were out of range. None of the abnormal vital signs were clinically significant, except for one participant who, however, reported to feel well. During the course of the study, none of the participants was excluded due to abnormal vital signs. A detailed description of all abnormal vital signs can be presented on request.

Headache severity

A total of n = 16 participants reported headaches either prior or after fMRI assessment, mean NRS score was = 1.4, all NRS scores were < 5, thus none of the participants was excluded due to reported headache.

Electrocardiogram

For n = 3 participants abnormal ECG values were detected. In accordance with the study protocol, measurements were repeated. During the repeated measurements, no abnormal values were detected.

Physical examination

Information for all physical examinations was included in the source documentation at the study site, only medically relevant findings were recorded in the eCRF. For examinations at the Screening visit, medically relevant findings were also captured as medical history in the eCRF.

Deaths and other serious adverse events

No AEs leading to discontinuation or other significant AEs occurred during the study.

Adverse Events leading to discontinuation and other significant AEs

One subject discontinued study participation due to a panic reaction in the scanner.

Clinical laboratory evaluation

The following parameters were assessed: sodium, postassium, calcium, magnesium, total protein, albumin, glucose, creatinine, urea, bilirubin, AST, ALT, GGT, LDH, AP, CRP; hematology: leukocytes, granulocytes, neutrophils, eosinophils, basophiles, lymphocytes, monocytes, erythrocytes, thrombocytes, haematocrit, haemoglobin; oagulation: aPTT, INR.

Plasma concentration

Plasma concentrations for lamotrigine and ketamine are depicted in the table below. No significant differences in ketamine plasma concentration was found between the lamotrigine and ketamine group (T(44) = 1.62, p = 0.11). These results were already published by Gärtner et al. 2023.

Table S3.

Ketamine	
+0:55 h	PK: 108.59 ng/L ± 27.61
	LK: 94.26 ng/L ± 32.45
**Lamotrigine**	
+0:30 h	2998.17 mg/L ± 1316.80
+1:00 h	3509.76 mg/L ± 1153.81
+1:30 h	3986.10 mg/L ± 990.62

+2:55 h	3943.42 mg/L ± 928.82
+4:00 h	3791.87 mg/L ± 698.83

Notes: Time points refer to infusion onset for ketamine and oral administration for lamotrigine.

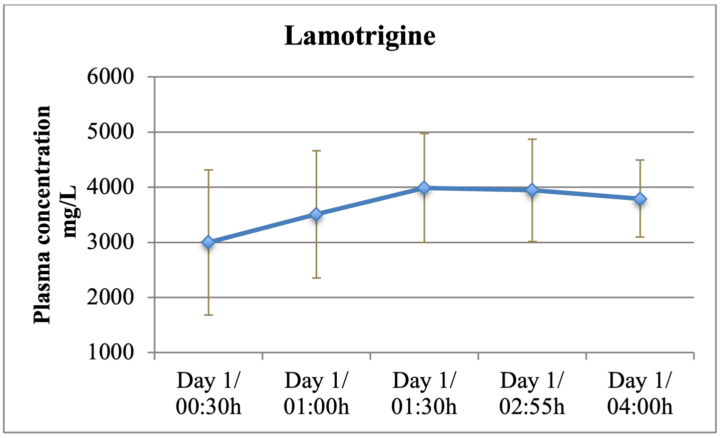

Figure S6. Plasma concentration of lamotrigine. Error bars depicting standard deviations. Time points refer to lamotrigine administration.

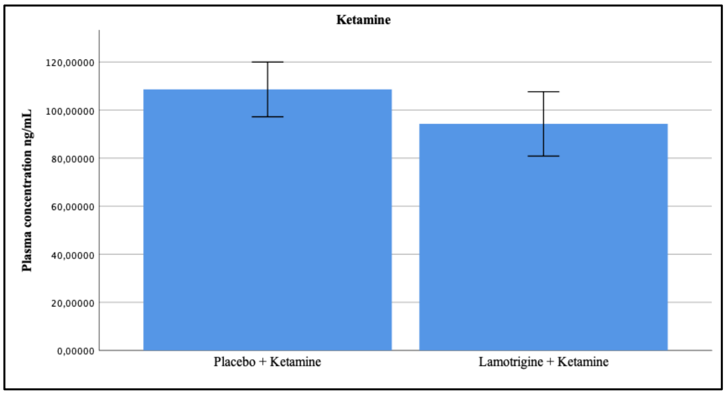

Figure S7. Plasma concentration of ketamine. Error bars depicting 95% confidence intervals.

Imaging acquisition

BOLD data was acquired using a 3 Tesla MRI scanner (PRISMA, Siemens Medical Systems, Erlangen, Germany) at the Berlin Center of Advanced Neuroimaging, employing a T2*- weighted gradient echo-planar imaging sequence (TR=2 s, TE=30 ms, flip angle=80°, voxel size=3x3x3 mm, matrix 64x64, 36 slices, FOV=192x192x143 mm, GRAPPA acceleration factor 2). An anatomical brain image was acquired with a 3D T1-weighted scan (Magnetization Prepared Rapid Acquisition Gradient Echo sequence, TE=3.03 ms, TR=2.3 s, 192 slices and FOV=256x256x192 mm). Moreover, to improve registration, a fieldmap was obtained employing a double-echo gradient echo field map

sequence (TR = 468 ms, TE = 4.92 / 7.38 ms, slices = 39, voxel size = 3x3x3 mm, flip angle = 60°, matrix = 64x64, FOV = 192x192x140).

Preprocessing and first-level analyses of fMRI data

Preprocessing. T1 anatomical data were biasfield corrected and aligned to the MNI152 standard space using linear alignment via FSL FLIRT with 12 degrees of freedom and subsequently refined non-linearly as implemented in FSL FNIRT. The processing of the functional brain images included correction for participant head motion, correction for EPI distortions using fieldmap data, and a 5 mm FWHM spatial smoothing. To identify and correct for more subtle effects of head motion FSL's MELODIC (Beckmann & Smith, 2004, 2005) was used to extract independent data components followed by ICA-AROMA (Pruim et al., 2015a, 2015b) to identify and remove secondary effects of head motion. Finally, a temporal 0.01 Hz high-pass filter was applied to remove scanner drifts. Furthermore, a transformation from the functional space to the T1 anatomical space using FSL Boundary Based Registration was obtained. Eventually, the transformation was combined with the T1 to MNI152 registration to transfer the functional data from the individual's native space to the MNI152 standard space.

First-level. A General Linear Model implemented in FEAT was used on the preprocessed participant-level data to obtain the contrasts of interest. First, the onsets of the respective task conditions were calculated from the log files and subsequently convolved with a double-gamma HRF to model the task-specific BOLD signal changes. The contrast of interested consisted of the affective condition > neutral condition contrast only. Thereafter, an estimate of the fit between the task regressors and the BOLD time series were obtained for each voxel, ultimately resulting in a map of statistically significant activations. Multiple comparisons correction was performed at the cluster level using Gaussian Random Field Theory (voxel: $z > 3.1$ uncorrected; cluster: $p < .05$, FWE-corrected). The resulting activation maps were used in subsequent group-level analyses. Moreover, to investigate functional coupling between regions of interest and the rest of the brain during the task, a psychophysiological interaction analysis was conducted. The respective regressor was acquired by forming the interaction term of the time series of the voxel with the best fit to the task contrast of interest (AC > NC) in each ROI as the seed and the task regressor of interest (AC). Applying this new interaction regressor using the General Linear Model described above resulted in a connectivity map of regions significantly correlated with the seed region as a function of task condition.
